# Synovial fluid extracellular vesicles as arthritis biomarkers: the added value of lipid-profiling and integrated omics

**DOI:** 10.20517/evcna.2024.14

**Published:** 2024-06-13

**Authors:** Laura Varela, Chris H.A. van de Lest, P. René van Weeren, Marca H.M. Wauben

**Affiliations:** ^1^Division Equine Sciences, Department of Clinical Sciences, Faculty of Veterinary Medicine, Utrecht University, Utrecht 3584 CM, the Netherlands.; ^2^Division Cell Biology, Metabolism & Cancer, Department of Biomolecular Health Sciences, Faculty of Veterinary Medicine, Utrecht University, Utrecht 3584 CM, the Netherlands.

**Keywords:** Arthritis, extracellular vesicles, exosomes, synovial fluid, biomarkers, lipidomics, horse, neutrophils

## Abstract

Arthritis, a diverse group of inflammatory joint disorders, poses great challenges in early diagnosis and targeted treatment. Timely intervention is imperative, yet conventional diagnostic methods are not able to detect subtle early symptoms. Hence, there is an urgent need for specific biomarkers that discriminate between different arthritis forms and for early diagnosis. The pursuit of such precise diagnostic tools has prompted a growing interest in extracellular vesicles (EVs). EVs, released by cells in a regulated fashion, are detectable in body fluids, including synovial fluid (SF), which fills the joint space. They provide insights into the intricate molecular landscapes of arthritis, and this has stimulated the search for minimally invasive EV-based diagnostics. As such, the analysis of EVs in SF has become a focus for identifying EV-based biomarkers for joint disease endotyping, prognosis, and progression. EVs are composed of a lipid bilayer and a wide variety of different cargo types, of which proteins and RNAs are widely investigated. In contrast, membrane lipids of EVs, especially the abundance, presence, or absence of specific lipids and their contribution to the biological activity of EVs, are largely overlooked in EV research. Furthermore, the identification of specific combinations of different EV components acting in concert in EVs can fuel the definition of composite biomarkers. We here provide a state-of-the-art overview of the knowledge on SF-derived EVs with emphasis on lipid analysis and we give an example of the added value of integrated proteomics and lipidomics analysis in the search for composite EV-associated biomarkers.

## INTRODUCTION

Movement is a fundamental hallmark of life, and the emergence of skeletal joints, particularly the diarthrodial joint, serves as a poignant example of how intricately movement is intertwined with the structure of the synovial joint^[1]^. However, genetic switches influencing joints, especially the knee, have triggered hereditary traits that affect millions worldwide^[2]^, and the “design” of the diarthrodial joint makes it vulnerable to conditions like arthritis^[2]^. Arthritis is not exclusive to humans. In fact, arthritis is widespread in the animal kingdom. Among animals affected by this disorder, there are companion animals such as cats, dogs, and horses, but also wild animals like bears, hyenas, bison, chimpanzees, gorillas, lions, foxes, and more^[3-5]^. Although arthritis encompasses a wide variety of inflammatory disorders, all types of arthritis lead to a common pathological outcome in which the joint’s structural integrity is affected and damage to one or more components of the joint (synovial membrane, cartilage, subchondral bone, and eventually structures such as menisci and ligaments) is present^[6,7]^. Currently, no definitive cure halts or reverses the progression of arthritis, and anti-inflammatory drugs and analgesics are commonly used to manage symptoms^[8,9]^. Furthermore, conventional diagnostic methods often fail to detect subtle early symptoms, and a diagnosis is often made at late stages when the damage is already substantial, thereby delaying therapeutic procedures and affecting their effectiveness^[10,11]^. Therefore, there is an urgent clinical need to identify biomarkers for the early stages of joint disease that would enable arthritis endotyping, allowing for tailored treatment strategies and timelier therapeutic interventions aimed at decelerating disease progression.

The search for such precise diagnostic tools has sparked an interest in extracellular vesicles (EVs). EVs are small lipid bilayer-enclosed particles containing a wide variety of different biological cargo molecules that are released by cells in a regulated fashion^[12]^. The clinical potential of EVs for diagnosis became clear when it was established that EV-mediated cell-cell communication can act over long distances, whereby EVs travel via body fluids and can pass epithelial and endothelial barriers, e.g., the blood-brain barrier^[13]^. Extracellular vesicles can be sourced from body fluids, including urine, saliva, blood, and synovial fluid (SF)^[14]^, which facilitates the search for non-invasive or minimally invasive EV-based diagnostics by monitoring changes in EV cargos^[15]^. As such, the molecular analysis of EVs in SF might provide crucial insights into the intricate molecular landscapes of arthritis. Despite the large interest in deciphering EV-associated proteins and RNA molecules, there is a void in understanding how specific EV components are assembled and act in concert in EVs. Furthermore, the role of the many lipids that can be found in the membranes of EVs has been largely overlooked in EV research thus far. In contrast to the classic images in scientific articles of EVs, which depict heterogeneous protein cargo and RNA composition in the presence of a homogenous lipid bilayer, the lipid bilayer, as such, is much more diverse. Hence, it can be anticipated that the abundance, presence, or absence of specific lipids contributes explicitly to the biological activity of EVs and that detailed lipid profiling of EVs, especially SF EVs in the case of joint diseases, can contribute to the identification of EV-based biomarkers for arthritis diagnosis, prognosis, or progression.

## INFLAMMATORY JOINT DISORDERS: THE ARTHRITIS SPECTRUM

The spectrum of arthritis includes many different joint disorders with distinct pathophysiological mechanisms, e.g., juvenile arthritis, gout, septic arthritis, ankylosing spondylitis, osteoarthritis (OA), rheumatoid arthritis (RA), axial spondyloarthritis, and psoriatic arthritis; the latter two are collectively termed “spondyloarthritis” (SpA). Below, we provide a short description of the most prominent members of the arthritis spectrum, i.e., OA and the autoimmune arthritis types, RA and SpA [Table 1].

**Table 1:** Summary chart for osteoarthritis, rheumatoid arthritis, and spondyloarthritis

**Type of arthritis**	**Key characteristics**	**Risk factors**	**Pathological features**	**Common treatments**
Osteoarthritis	Degenerative, affects mainly elderly, involves cartilage damage and synovial inflammation	Aging, obesity, trauma, mechanical stress, genetic predisposition	Osteophyte formation, cartilage degradation, synovial inflammation	Pain management, lifestyle changes, surgical interventions
Rheumatoid arthritis	Autoimmune, persistent joint pain, swelling, stiffness; affects synovium leading to tissue damage	Genetic factors, environmental triggers	Synovial joint inflammation, tissue damage, increased vascularity	DMARDs (disease-modifying antirheumatic drugs), biologicals
Spondyloarthritis	Includes PsA, AS, arthritis with IBD, ReA; joint inflammation, skin/gut/eye manifestations, seronegative	Overlaps with RA risk factors, complex genetic and environmental interactions	Asymmetrical synovial joint inflammation, increased neutrophil infiltration	NSAIDs, biologic agents, physical therapy

PsA: Psoriatic arthritis; AS: Ankylosing spondylitis; IBD: Inflammatory bowel disease; ReA: Reactive arthritis; RA: Rheumatoid arthritis; NSAIDs: Non-steroidal anti-inflammatory drugs.

### Osteoarthritis

In humans, OA impacts nearly 500 million people worldwide, equivalent to about 7% of the global population^[16]^. This prevalence is especially high in the elderly^[17]^. As a degenerative condition, OA stands out as a noticeable affliction among mammals^[18]^. However, its precise pathogenesis remains a puzzle. Though OA may not be immediately life-threatening, it severely hampers the quality of life, and with the strongly increasing prevalence, it imposes an economic burden on the healthcare budget^[19,20]^.

OA predominantly results from the cumulative effects of aging, wear and tear, traumatic injuries, genetic predisposition and/or athletic impact^[21]^. The resulting injuries release mediators that initiate a series of inflammatory pathways that lead to cartilage damage. Simultaneously, synovial inflammation is evident in OA joints to a certain extent, correlating with radiographic findings and pain progression^[7]^. Numerous risk factors, including age, obesity, trauma, and mechanical stress, influence the development of OA, possibly by altering synovial biology. Additionally, elements like mitochondrial dysfunction, cytokines, and metabolites in the synovium amplify synovial inflammation by activating synovial cells^[22]^. Pathologically, OA showcases a myriad of alterations, e.g., osteophyte formation, degradation of articular cartilage, subchondral bone thickening, synovial inflammation, ligament degeneration, changes in knee menisci, and joint capsule hypertrophy^[23]^.

### Autoimmune arthritis

The predominant members of autoimmune arthritis are RA and SpA. Rheumatoid arthritis is a persistent condition marked by joint pain, swelling, and stiffness. It predominantly affects areas like the knees, hands, feet, wrists, and others. Rheumatoid arthritis is an autoimmune inflammatory disorder hallmarked by inflammation of the synovium that, over time, can result in tissue damage, persistent pain, and structural deformities^[24]^. Globally, RA affects roughly 1% of the human population. The onset of RA is believed to stem from complex interactions between genetic factors and environmental triggers, leading to an imbalance in immune tolerance^[25]^. The exact cause of RA remains elusive, yet significant factors linked to its progression have been pinpointed^[25]^. Spondyloarthritis encompasses a range of arthritic diseases, including psoriatic arthritis (PsA), ankylosing spondylitis (AS), arthritis associated with inflammatory bowel disease, and reactive arthritis (ReA)^[26]^. In Western Europe, SpA prevalence ranges from 0.3% to 2.5%^[26]^. The SpA subtypes share several clinical and immunological features, such as joint inflammation affecting both the peripheral and axial skeleton, manifestations in the skin, gut, and eyes, and a seronegative status indicating the absence of diagnostic autoantibodies^[27]^. A hallmark of these diseases is synovial joint inflammation, which often exhibits an asymmetrical pattern^[28]^. Given the overlap in symptoms and characteristics, accurately classifying patients based on clinical phenotyping into specific SpA categories is complex, especially in the early stages of the disease. This challenge might become easier to tackle with improved molecular endotyping, i.e., describing the underlying molecular mechanisms that drive disease phenotypes^[29]^.

Both RA and SpA patients display increased vascularity in the synovia and infiltration by polymorphonuclear cells, especially neutrophils, which belong to the innate immune system but can also modulate adaptive immune responses^[30,31]^. The strongly elevated presence of neutrophils seen in SpA SF may contribute to the onset of pathogenic Th17 responses^[32]^. Unlike SpA, in RA, there is also an increased infiltration of T cells and B cells into the synovial tissue^[33]^, which are instrumental in determining the trajectory of the disease.

## ONE HEALTH APPROACH: THE EQUINE PATIENT AS A MODEL

The large and urgent clinical need for early-stage arthritis biomarkers is not restricted to humans. Osteoarthritis is common in many mammalian species and is a big issue in dogs, cats, and especially horses. In the latter species, OA accounts for 60% of all cases of lameness^[18,34]^_._ Equine OA is a critical issue for animal welfare, which translates into reduced mobility and lameness, discomfort, poor performance, and early retirement, thereby also inflicting significant financial losses on the equine industry^[35]^. Horses have a biochemical composition of joints that is surprisingly similar to that of humans and, unlike the classic laboratory species, carry higher biomechanical loads than humans, which makes the species an interesting translational model^[36-38]^. Besides the translational aspects for human and equine OA patients, experimental arthritis models have also been developed using the horse as an animal model for the study of human arthritis, such as the equine groove model^[39,40]^ and the well-established lipopolysaccharide (LPS) model to study acute (inducible) synovitis^[41,42]^. With the equine models, we can effectively employ techniques such as controlled exercise (e.g., use of a treadmill), quantitative gait analysis as a measure of joint functionality, and imaging methods such as radiography, computed tomography (CT), magnetic resonance imaging (MRI), and arthroscopy. Moreover, the easiness with which arthrocentesis can be performed and the relative abundance of SF allow for minimally invasive taking of samples for SF analysis.

## THE SYNOVIAL JOINT

**Figure 1:**
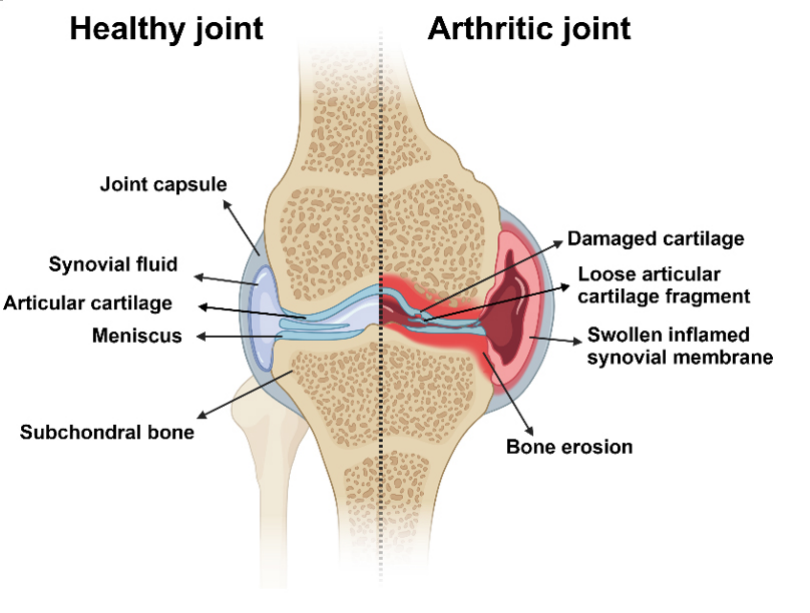
Schematic cross-section of the synovial joint in a healthy and inflamed state. *Created with BioRender.com.*

In a synovial joint, the primary articular components include the articular cartilage with underlying subchondral bone, synovial cavity, synovial fluid, synovial membrane, ligaments, tendons, and menisci (in specific joints like the knee) [Figure 1]. These components work in concert to provide smooth, coordinated movement and stability to the joint. The hyaline articular cartilage lines the articulating surfaces of bones in synovial joints and is populated almost exclusively by chondrocytes. This strong and flexible connective tissue protects the underlying subchondral bone and serves as a shock absorber^[43]^. Moreover, cartilage provides an extremely smooth surface with a low coefficient of friction, facilitating efficient joint movement. Chondrocytes are responsible for the formation and the maintenance of the cartilage extracellular matrix (ECM) by releasing proteoglycans and collagens that form a meshwork of collagenous fibers that together endow cartilage with its strength, resilience, and flexibility, all of which are necessary for the cartilaginous tissues in joints to function correctly^[43]^. In addition to chondrocytes, 0.1%-1% of the total cartilage cells are articular cartilage stem/progenitor cells (ACPCs)^[44,45]^. The articular capsule consists of the outer fibrous layer and the internal synovial membrane, and encloses the entire joint. The synovial membrane comprises two primary layers: the lining layer, which directly communicates with the articular cavity but lacks blood vessels and a basement membrane, and the sub-lining layer, a rather loose connective tissue network interwoven with blood vessels and sparse cells^[46]^. Synovial fibroblasts and macrophages, which are tissue-resident cells, support the joint microenvironment. These cells play pivotal roles in maintaining tissue equilibrium and joint homeostasis and modulating local inflammatory responses. Fibroblast-like synoviocytes (FLS) produce lubricating molecules like hyaluronan and lubricin, as well as collagen and proteoglycans. Tissue-resident macrophages are evenly dispersed across the lining and intermittently in the sub-lining^[47]^. These macrophages are essential for the regulation of tissue physiology through their phagocytic actions and the release of pro- or anti-inflammatory mediators^[46]^. Synovial fluid (SF) is a refined ultrafiltrate of blood plasma, which is enriched by the products from the cellular components of the synovial membrane as it passes through this structure^[47]^. This fluid serves multiple roles, including biomechanical, metabolic, and regulatory ones. Central to its function, SF maintains the rheological properties of articular cartilage, acting as a lubricant to minimize friction between cartilage interfaces and between the synovium and cartilage. Additionally, it is vital for nutrient transport, metabolite exchange, and waste elimination in synovial tissues^[47]^. During homeostasis, the fluid is replete with soluble molecules such as morphogens, growth factors, and cytokines, as well as with more complex biological colloidal structures, which are all essential for intercellular communication within the joint.

In an inflammatory state beyond the already mentioned soluble molecules, there is an increase in lipoprotein particles, EVs, and infiltrating cells, which depends on the type of arthritis^[48-50]^. Recent studies on SF and synovia from RA patients have shown a pronounced enrichment of EVs derived from fibroblasts, T cells, monocytes, platelets, and neutrophils^[51]^. In contrast, OA SF predominantly features EVs from T cells, macrophages, B cells, natural killer cells, dendritic cells, and joint-resident cells^[52]^. Interestingly, the size and concentration of EVs in SF appear to show minimal variance between OA patients and healthy individuals^[53,54]^. This suggests that possible pathological distinctions may lie in the particular EV subtypes from SF with unique endotype signals.

## EXTRACELLULAR VESICLES

Research on unveiling the roles of so-called extracellular vesicles (EVs) in physiological and pathophysiological processes is thriving, since EVs are perceived as rich biomarker reservoirs for diagnostics and as holding great therapeutic application prospects^[55]^.

**Figure 2:**
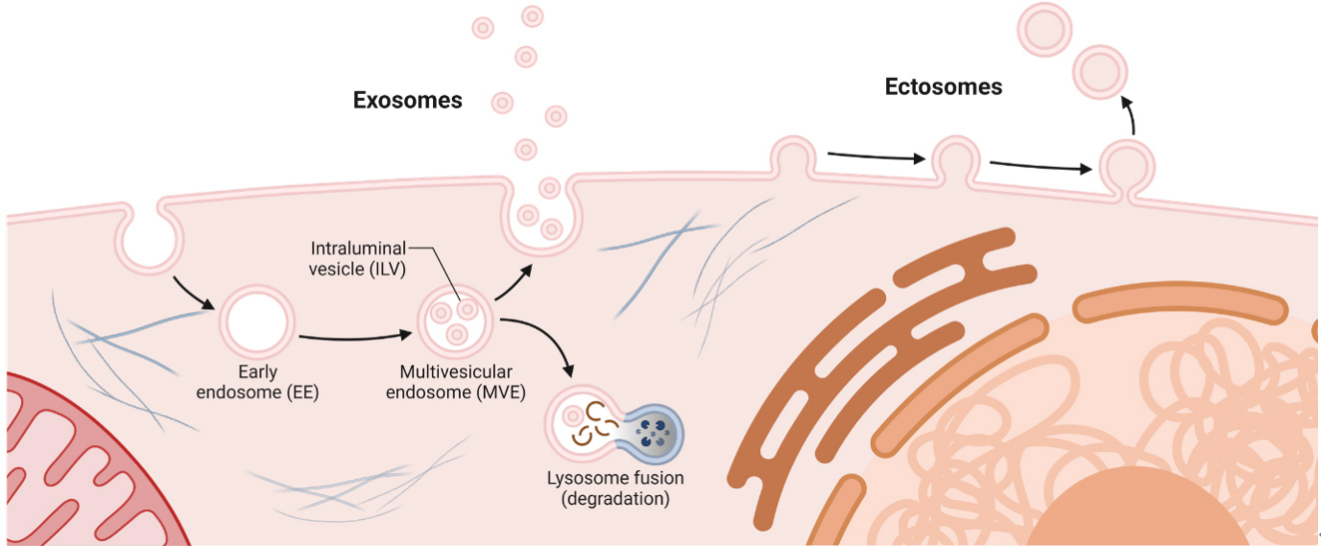
Schematic representation of the basic principles of EV biogenesis. *Adapted from the “Extracellular Vesicles” template by BioRender.com (2023).*

EVs are defined as lipid bilayer-delimited spherical structures that are released by cells and involved in cell-cell communication^[55,56]^. EVs can convey a multitude of messages via different messenger molecules, ranging from DNA, RNA, proteins, and metabolites to lipids^[57-59]^. A wide collection of EV-cargo data is currently available in online databases like ExoCarta^[60]^, Vesiclepedia^[61]^, and EVpedia^[62]^. EVs can be classified into exosomes, which can be up to 200 nm in size, and ectosomes, which vary broadly in size, spanning from as small as exosomes to several micrometers. This classification hinges not on size but on their biogenesis pathways: exosomes originate from endocytic compartments, whereas ectosomes are formed through budding from and blebbing of the plasma membrane^[63]^ [Figure 2].

Exosome production originates from multivesicular endosomes (MVEs), where inward budding forms intraluminal vesicles (ILVs). During ILV formation, specific proteins and lipids embedded in the delimiting membrane are selectively incorporated, and cytosolic cargoes are sequestered within the lumen of ILVs. MVEs either merge with lysosomes for content degradation or with the plasma membrane, thereby releasing ILVs as exosomes into the extracellular milieu^[12]^. Exosome biogenesis roughly follows two main pathways: either the ESCRT (endosomal sorting complex required for transport)-dependent or the ESCRT-independent pathway^[12]^. The ESCRT machinery is essential for sorting ubiquitinated proteins into MVEs and facilitating membrane budding and fission. ESCRT-0 recognizes and sequesters ubiquitinated proteins, guiding them to the endosomal membrane. ESCRT-I and -II induce membrane curvature and cluster ubiquitinated proteins, while ESCRT-III drives the membrane scission process, leading to intraluminal vesicle formation^[12]^. The ESCRT-independent route relies on lipids, particularly on ceramide. Conversion of sphingomyelin to ceramide by neutral sphingomyelinases (nSMases) can drive the inward budding of endosomal membranes, resulting in ILV formation within MVEs^[64]^. Numerous proteins, such as SNAREs and Rab family GTPases, are involved in exosome secretion. Depending on the cell type and status of the cell, canonical exosome biogenesis pathways can converge with different intracellular pathways, e.g. the autophagy pathway resulting in the fusion between MVEs and autophagosomes, which contributes to the heterogeneity of exosomes released by cells^[12,65]^.

Ectosome biogenesis is influenced by the lipid and protein interactions in the plasma membrane, with specific lipids like sphingolipids and cholesterol playing pivotal roles. The biophysical properties of the plasma membrane, including lipid order, lateral heterogeneity, and plasma membrane lipid domains, as well as specialized membrane structures, e.g., cilia and elongated cell protrusions, are crucial determinants in ectosome formation^[66]^.

Notably, both plasma membrane-derived EVs and endosomal ILVs bud away from the cytosol. Hence, their membrane orientation is identical to that of the cell surface, which exposes the extracellular domains of the transmembrane proteins and encloses cytosolic components^[12]^. Some proteins, such as the transmembrane 4 superfamily proteins (e.g., CD9, CD63, and CD81), heat shock proteins (e.g., Hsp90. Hsp70), ALIX, TSG101, and cytoskeleton elements such as actin, have been defined as generic EV-markers^[67]^. Besides these generic markers, EVs also carry proteins like integrins and the previously mentioned GPI-anchored proteins. Furthermore, EVs contain specific protein signatures of the cell of origin, which vary depending on the cell type, as well as the status of the cell^[68-70]^. Overall, it is difficult to discriminate between exosomes and small ectosomes once these EVs are released in the extracellular space. Although this complicates the determination of EVs isolated from body fluids, differences in membrane composition, encompassing both proteins and lipids, as well as other types of cargo molecules, have been identified^[71]^. In the section “LIPIDS: THEIR IMPORTANCE FOR EV RESEARCH,” we delve deeper into the EV-lipidome.

## SYNOVIAL FLUID EVs: BIOMARKERS FOR INFLAMMATORY JOINT DISORDERS?

**Figure 3:**
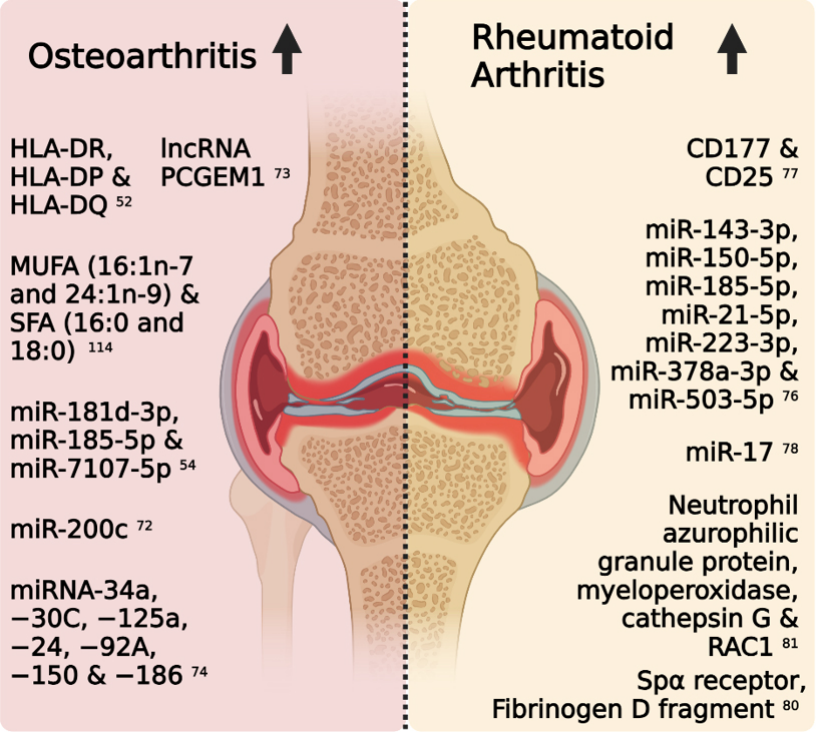
Schematic state-of-the-art figure of EV-associated potential biomarkers that increase during OA and RA. HLA: Human Leukocyte Antigen; lncRNA: long non-coding RNA; PCGEM1: Prostate Cancer Gene Expression Marker 1; MUFA: monosaturated fatty acid; SFA: saturated fatty acid; miR: microRNA; RAC1: Ras-related C3 botulinum toxin substrate 1; OA: osteoarthritis; RA: rheumatoid arthritis. *Created with BioRender.com*.

In recent years, the significance of EVs in OA research has become increasingly apparent [Figure 3]. A study by Withrow *et al.* demonstrated that EVs isolated from the SF of OA patients exhibited a pronounced enrichment of miR-200c in comparison to their healthy counterparts^[72]^. This increase in miR-200c might be associated with the degradation of articular cartilage, positioning EV-associated miR-200c as a potential early biomarker for OA. Complementing this finding, Kolhe *et al.* unveiled gender-specific variations in the miRNA content of EVs, e.g., miR-181d-3p, miR-185-5p, and miR-7107-5p^[54]^. These miRNAs influence genes associated with the female estrogen signaling pathway, modulating the expression of ER-α (estrogen receptor-α), ER-β (estrogen receptor-β), and CYP19 (aromatase cytochrome P450), as well as targeting the CREB-binding protein in human joint chondrocytes. Hence, the authors suggest that the increased incidence of OA in post-menopausal women is related to reduced estrogen levels, which could affect the secretion of EVs and the composition of their miRNA cargo^[54]^. Furthermore, EVs carrying HLA-DR, -DP, and -DQ in OA SF surpass that in the blood by 25-50 times, indicating that infiltrating immune cells might be the dominant contributors to the SF EV pool^[52]^. In addition, lncRNA PCGEM1 has been found in OA SF EVs^[73]^. PCGEM1 is known to target miRNA-770, foster synoviocyte proliferation, and display distinct expression levels depending on the disease state of the joint. These levels not only varied between OA patients and healthy individuals but also between the progressive and initial stages of OA^[73]^. Similarly, the miRNA and protein profiles of EVs extracted from OA-SF mirrored those sourced from aging chondrocytes. After undergoing senolytic treatment, a range of miRNAs -- specifically miRNA-34a, -30C, -125a, -24, -92A, -150, and -186, all pivotal in chondrogenesis -- revealed significant changes^[74]^.

The clinical diagnosis of RA necessitates a minimum of one clinically swollen joint and a score of 6 out of 10 based on specific criteria^[75]^. Points are accrued from joint evaluations via physical or imaging methods (up to 5 points), elevated rheumatoid factor and anti-citrullinated protein antibody levels (2 or 3 points depending on the severity), and factors like increased acute phase reactant response and symptom duration of at least six weeks (1 point each)^[75]^. In the majority of early RA cases, timely diagnosis and intervention can halt the progression of joint deterioration in approximately 90% of patients^[10]^. Consequently, the incorporation of potential complementary criteria may enhance the diagnostic accuracy for RA through an optimized composite biomarker panel, which might be further improved through the integration of EV-based biomarkers. Likewise, EV research in RA has proved instrumental to the determination of molecular characteristics that differentiate RA from OA or the normal phenotype [Figure 3]. In patients with RA, SF contains EVs at concentrations higher than SF from OA patients^[76,77]^. Although the amount of protein of SF EVs of RA exceeds that in OA, in both SF sources, CD177 (neutrophils), CD14 (monocytes), CD62E (activated epithelium), and CD25 (T-reg cells) have been discovered^[78]^. EVs originating from RA SF demonstrated an increased prevalence of all these proteins, notably with a higher proportion of CD25, indicative of increased T-cell activation, and CD177, indicating increased neutrophil activation. These findings corroborate the prominent infiltration of T cells and neutrophils in the joints of RA patients^[78]^. Furthermore, the dominance of neutrophil-derived EVs in SF of RA was also confirmed by Foers *et al.*, describing 45 proteins significantly elevated in SF EVs of RA patients with high levels of inflammation compared to low levels of inflammation, while 135 proteins were significantly elevated compared to SF EVs of OA patients, with proteins associated with "neutrophil degranulation" (42/135 proteins) being significantly enriched in SF EVs of RA patients with high levels of inflammation^[77]^. Overall, RA SF EVs prominently feature markers of the neutrophil lineage and their granule proteins, e.g., the neutrophil azurophilic granule protein, myeloperoxidase (MPO) detected at concentrations nearly nine times higher in severely inflamed RA joints compared to their mildly inflamed counterparts^[77]^. Similarly, post-translationally modified proteins, such as citrullinated and carbamylated proteins^[79]^, have been strongly associated with RA and have been proposed as biomarkers. Intrinsically, EVs have been shown to be associated with citrullinated proteins, including fibrinogen D fragment, Sp alpha (like CD5 antigen protein) receptor, and fibrinogen beta chain precursor, among others^[80]^. Furthermore, cathepsin G (CTSG) and Ras-related C3 botulinum toxin substrate 1 (RAC1), which are pivotal for T cell responses, FLS activation, and ROS generation, were shown to be upregulated in RA-affected joints with high and low levels of inflammation, respectively^[77]^.

Interestingly, functional analysis of EVs isolated from RA patients, both from the circulation and SF, showed that these EVs can influence T cell activity, disrupt the Th17/T-reg balance, and can modify inflammatory cytokine concentrations^[81]^. Notably, elevated levels of miR-17 in circulating EVs of RA patients have been found to impede T-reg differentiation by curbing TGF-β receptor II expression^[81]^. Similarly, increased levels of inflammatory-associated miRNAs have been detected in SF EVs from RA, such as miR-143-3p, miR-150-5p, miR-185-5p, miR-21-5p, miR-223-3p, miR-378a-3p, and miR-503-5p, through the targeting of IGF1R (insulin-like growth factor 1 receptor), VEGFA (vascular endothelial growth factor A), and BCL1 (an apoptosis regulator)^[76]^.

## LIPIDS: THEIR IMPORTANCE FOR EV RESEARCH

Importantly, compared to the analyses of the protein and RNA composition of EVs, the analysis of the lipid composition of EVs lags behind. Consequently, as also illustrated in Figure 3, lipid components of EVs are underrepresented in the list of EV-associated potential biomarkers in arthritis. Lipids are essential components of the EV architecture, and the lipid bilayer could potentially offer a unique perspective into the origin, function, and possible pathological alterations of EVs in joint diseases. Furthermore, evidence is accumulating that EV-associated lipids may be actively involved in the functional modulation of target cells by EVs^[82,83]^. Hence, the exploration of the EV lipidome provides an exciting avenue for future research, shedding light on the complex relationship between lipids and other EV components and the role of EV-associated lipids in intercellular communication and in (patho)physiological processes.

### Lipid distribution in cell membranes

For the function of membranes, the asymmetric distribution of lipids in membranes is essential. For instance, phosphatidylserine (PS) is concentrated on the cytosolic side of the plasma membrane of viable cells^[84]^. Upon translocation to the external leaflet of the plasma membrane mediated by protein kinase C, lipid flippases and floppases, PI3-kinases, fluctuations in Ca-levels, or cell stress (e.g., during apoptosis), this lipid is recognized as “eat-me” signal, integral for phagocyte recognition^[84]^. Notably, the species PS(18:0/18:1) plays a crucial role in regulating cholesterol distribution in lipid bilayers, retaining cholesterol in the inner leaflet (where PS is typically located^[85]^), and preventing its redistribution to the outer leaflet^[86]^. Additionally, PS(18:0/18:1) induces phase separation in cholesterol-containing bilayers, shields cholesterol from oxidation, and influences membrane curvature, suggesting its significance in the assembly of membrane nanodomain structures like caveolae^[86]^, which could influence EV biogenesis and delivery. Similarly important, phosphatidylinositol (PI), though a minor phospholipid, is also primarily located in the cytosolic layer of cell membranes^[84]^. Lipid kinases can modify PI by adding phosphate groups at different positions on the inositol ring. These modified lipids become biological regulators of signals as binding sites that attract specific proteins from the cytosol to the membrane^[87]^. Glycolipids, which are lipid molecules attached to carbohydrate moieties, also exhibit significant membrane distribution asymmetry. They are found exclusively in the non-cytosolic layer of the lipid bilayer and are commonly incorporated into lipid rafts^[88,89]^.

### Lipid composition of EVs

**Figure 4:**
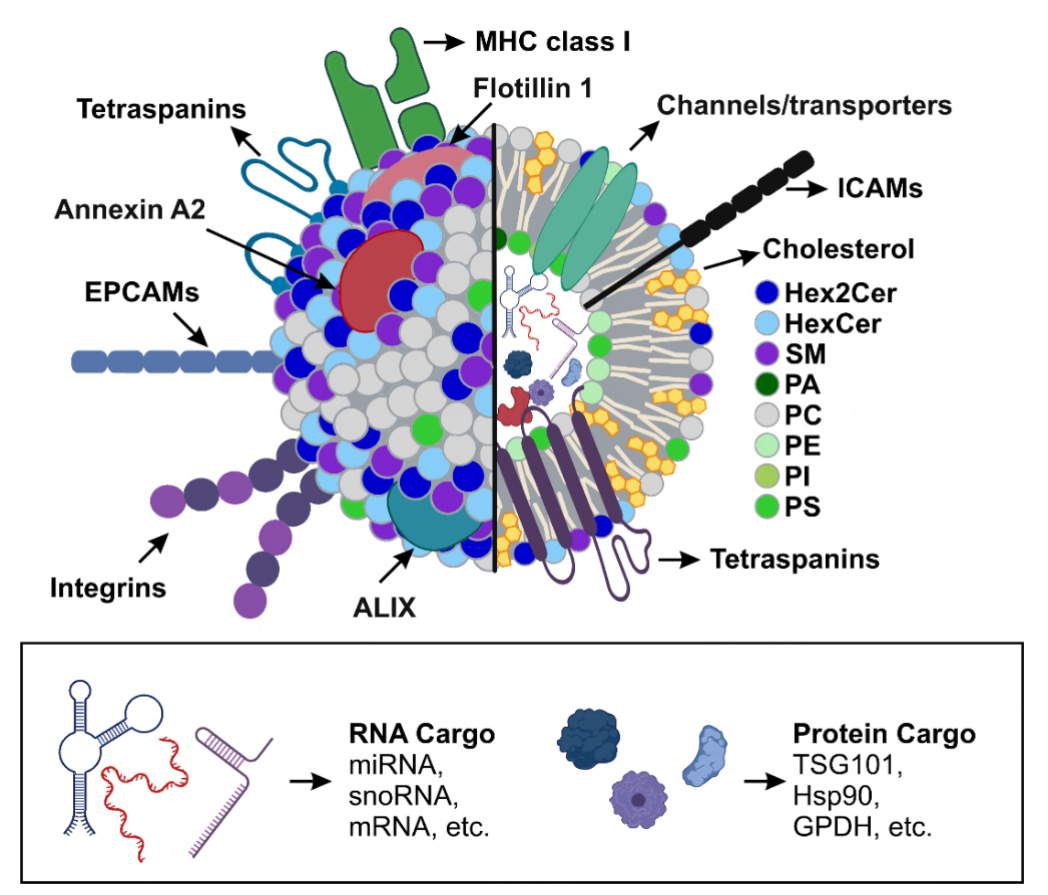
Illustration of a generic mammalian EV exhibiting its structural components. The lipid bilayer comprises diverse lipid species with sphingolipids, such as Hex2Cer, HexCer, SM, and glycosphingolipids like PA, PC, PE, PI, and PS, while enriched in cholesterol. Embedded within the lipid bilayer are various transmembrane proteins, e.g., tetraspanins, integrins, channels/transporters, adhesion proteins, and receptors. Furthermore, the EV encapsulates specific cargo, such as RNA and proteins. Hex2Cer: dihexosylceramide; lactosylceramide; HexCer: hexosylceramide; SM: sphingomyelin; PC: phosphatidylcholine; PI: phosphatidylinositol; PS: phosphatidylserine; PA: phosphatidic acid; PE: phosphatidylethanolamine; MHC: major histocompatibility complex; ICAMs: intercellular adhesion molecules; EPCAMs: epithelial cell adhesion molecule; Alix: apoptosis-linked gene-2-interacting protein X; miRNA: microRNA; snoRNA: small nucleolar RNA; mRNA: messenger RNA; TSG101: tumor susceptibility gene 101; Hsp90: heat shock protein 90; GPDH: glyceraldehyde 3-phosphate dehydrogenase. *Adapted from BioRender.com.*

Drawing attention to the lipid composition of EVs, it is noteworthy that in most published generic EV figures, specific EV-associated proteins and RNA biotypes are depicted while the lipid bilayer is often represented as a uniform structure. However, studies on the lipid composition of EVs from various cell types generated insight into their lipid profiles, and provided generic features that characterize the lipidome of EVs [Figure 4]. Of course, as for EV-associated proteins and RNA biotypes, the actual lipid composition of EVs can vary substantially depending on the originating cell type and its activation status^[90-92]^, thereby affecting biodistribution, targeting, and function. Overall, a detailed examination of the lipid composition in EVs showed that they are generally enriched up to 3 times in cholesterol, sphingomyelin (SM), glycosphingolipids, and PS compared to cells^[93]^. PS, recognized as the “eat me” signal when localized in the outer leaflet, leads to the swift clearance of EVs from circulation by phagocytic cells^[91]^. In contrast, most EV preparations have a lower concentration of phosphatidylcholine (PC) and PI than cells, while the PE content is comparable in both entities^[85]^. The presence of PI phosphate derivatives, PIPs, has not been thoroughly investigated. However, Jin *et al.* demonstrated the presence of PI_4_P and PI_3_P in macrophage‐derived EVs and attributed modulatory properties to PI_4_P in EV biogenesis^[94]^. Interestingly, Bis(monoacylglycerol)phosphate (BMP), which constitutes about 15% of phospholipids in late endosomes, is notably scarce in EVs, being often present below detectable levels and, when detected, accounting for 0.8%-1.2% of the lipidome^[91]^. Several of these generic EV lipidic features have been associated with lipid rafts, such as SM, lysophospholipids, glycosphingolipids, and cholesterol^[66,95,96]^. Lipid rafts play pivotal roles in several vesicular processes, especially in vesicle formation (particularly MVE), cargo loading, and subsequent anchoring and fusion with target membranes, and the generic EV-associated protein flotillin is an integral membrane protein in lipid rafts^[96]^. Interestingly, the lipid composition of some EV types seems to bear a striking resemblance to that of lipid rafts. Whether this similarity bears functional importance or merely reflects the vesicle origination remains an open question^[97]^.

Importantly, cell type-specific variations in the EV-lipidome have also become apparent. For example, EVs derived from mouse oligodendrocytes, human prostate adenocarcinoma, B cells, and hepatocellular carcinoma, among others, are particularly rich in cholesterol. In most of these different EV types, PC is the second most abundant lipid, except for B cells and human prostate adenocarcinoma-derived EVs, which contain a higher concentration of SM over PC^[91]^. In contrast, oligodendrocytes-derived EVs have higher levels of ceramide (Cer) and a decrease in SM compared to human prostate adenocarcinoma-derived EVs^[90,98]^. The following sections delve deeper into specific disease-associated and neutrophil-associated EV lipids.

### Disease-associated changes in EV lipids

Recently, lipidomics profiling of EVs in cancer has unveiled disease-associated lipid profiles, e.g., EVs in breast cancer and murine melanoma contain up to 80%-90% PC, while pancreatic cancer EVs show a lipid composition of 60% PC and 30% SM^[99]^. Interestingly, melanoma-derived EVs from highly metastatic cells are noteworthy for their higher content of arachidonic acid (AA), which is linked to prostaglandin E2 production^[100]^. These EV membranes are more fluid due to the increased polyunsaturated lipids, which facilitate vesicle-cell fusion. In glioblastoma, stem-like cell-derived EVs, distinct Cer profiles have been observed compared to the cell of origin^[101]^. Lipidomic analysis of EVs from different human cell lines and colorectal cancer patients revealed distinct lipid signatures linked to cancer stage, i.e., primary cancer EVs had increased levels of PC 34:1 and PE 36:2, while metastatic conditions showed a decrease in these lipids but an increase in Cer(d18:1/24:1)^[102]^. These distinct signatures, reflective of the cancer stage, present potential avenues for biomarker development, as has been suggested for prostate^[103]^, pancreatic^[104]^, and non-small cell lung^[105]^ cancers.

Besides cancer, changes in lipid profiles in EVs have been observed in other diseases. During acute myocardial infarction, patients exhibit significant alterations in their plasma EV lipid profiles^[106]^. In these patients, there is a notable increase in the content of Cer and SM within EVs, which directly correlates with peaks in high-sensitivity troponin, a marker used to diagnose heart injury and acute coronary syndrome^[106]^. In the context of psoriasis, circulating EVs in the plasma present distinct lipid profiles compared to healthy blood donors. Interestingly, when these patients are treated with ustekinumab (an interleukin-23 and interleukin-12 inhibitor^[107]^), the lipid profile of their plasma EVs closely mirrors that of healthy individuals^[108]^. It is also interesting to know that adipose EVs also display a specific lipidomic signature [enriched in Cer, SM, and phosphatidylglycerols (PG)], which is informative of the obesity metabolic state^[109]^. Notably, brain-derived EVs are abundant in PS and ether-linked PS lipids, and EVs derived from Alzheimer's disease brains exhibit distinct lipid profiles compared to controls^[110]^. Moreover, external factors such as ethanol consumption can lead to differential effects on human plasma EV lipid profiles^[111]^. Ethanol intake causes a reduction in SM while increasing Cer levels in both sexes. However, in ethanol-intoxicated females specifically, there is an enrichment in phosphatidic acid (PA) and lysophosphatidylcholine (LysoPC) species in plasma EVs, signifying gender-specific variations in lipid classes^[111]^.

Hence, besides all currently ongoing efforts to define EV-associated protein and RNA signatures, defining specific EV-lipidome signatures could be an alternative or synergistic approach to the design of EV-based biomarkers.

### Functional effects of EV-associated lipids

To unveil the contribution of fatty acids (FAs) from phospholipids as signaling molecules, recent publications have shed light on EV-associated FA compositions. For example, in 3T3 fibroblast cell cultures, MVEs were shown to be enriched in phospholipid species with polyunsaturated fatty acids (PUFA) consisting of 4 to 7 double bonds, while in 3T3 fibroblast-derived EVs, main PUFAs were linolenic acid (18:3n-3, an omega-3 FA) and linoleic acid (18:2, an omega-6 FA)^[112]^. Furthermore, during inflammatory conditions such as LPS-induced acute lung injury, which results in an increase in the release of EVs into the alveolar space derived from macrophages, epithelial cells, and neutrophils, EVs carry a diverse range of lipid mediators derived from omega-3 and omega-6 PUFA^[113]^. Interestingly, in EV-enriched pellets from equine OA SF, elevated levels of saturated FA like 16:0 and 18:0 and monosaturated 16:1n-7 (a fairly rare FA) and 24:1n-9 were found, which have strong associations with inflammation and OA progression^[114]^.

The functional role of sphingolipids in EVs is multifaceted. In the biogenesis of EVs, enzymes like acid and neutral sphingomyelinases (a-SMase and n-SMase) can facilitate EV biogenesis by generating ceramide accumulation in the inner leaflet at the plasma membrane and within endocytic compartments^[115]^. a-SMase is predominantly associated with EVs with a budding type of release, i.e., ectosomes budding from the plasma membrane, under conditions like stress or receptor stimulation^[115]^. Certain inhibitors of n-SMase can enhance ectosome production; therefore, the regulation of EV secretion by n-SMases indicates that inhibiting these enzymes can shift the balance between exosome and ectosome release^[116]^. Importantly, inhibition of n-SMases results in EVs with distinct cargo composition, suggesting a compositional difference between exosomes and ectosomes^[116]^. Additionally, other sphingolipids, such as sphingosine-1-phosphate and galactosyl-sphingosine, have been identified as playing a role in cargo sorting and EV formation^[115]^.

Moreover, LAPTM4B (Lysosome Associated Protein Transmembrane 4B) plays a significant role in influencing the lipid composition and stability of exosomes. It can modulate their ether lipid and glycosphingolipid composition^[117]^. LAPTM4B regulates the mammalian target of rapamycin signaling (mTORC1), which modulates autophagy, cell proliferation, and apoptosis through its participation in several signaling pathways. LAPTM4B is sorted explicitly into intraluminal vesicles of MVEs (which can be released as exosomes) by the sphingolipid interaction motif (SLim) found in its third transmembrane domain of LAPTM4B.

Evidently, lipids play a pivotal role in the intricate landscape of EVs, not only shaping their architecture but also influencing cellular communication in health and disease. The unique lipid composition of EVs, distinct from their parent cells, underscores the potential of lipidomics in unraveling specific endotypes associated with various diseases. In addition, harnessing the synergy of other omic technologies holds the promise of unveiling composite biomarkers for diverse endotypes, providing a key to deciphering the interplay between lipids and EV function.

## LIPID PROFILING OF SYNOVIAL FLUID EVs: THE HORSE AS A MODEL

Since inflammation is common to all forms of arthritis^[7,10,118]^, the horse has been studied not only as a patient but also as a representative model for arthritis in humans. As described previously in the section *“One Health Approach: The Equine Patient as a Model,”* horses provide an exceptional translational model due to the biochemical composition similarities in their joints compared to that of humans and the relative ease of collecting large amounts of SF. The LPS model in horses, inducing an acute-yet transient-synovitis^[41,42]^, enables efficient tracking of the onset of inflammation and subsequent resolution, with the peak of inflammation being observed between 5-8 h and the initiation of the resolution process becoming evident from 24 h onward^[119,120]^. Analysis of EVs derived from SF at various stages, i.e., healthy joints, at the peak of inflammation, at the onset of the resolution, and later during the resolution stage, showed a strong increase in EV number during the early phases of acute synovitis, which strongly decreased during the resolution phase^[121]^. Remarkably, during the peak of inflammation, the lipid profile of SF EVs was substantially changed compared to healthy SF-derived EVs, with a strong relative increase in HexCer (hexosylceramide, widely known as glucosylceramide). This inflammation-induced change in EV lipidomic profile remained quite similar during the resolution phase for a prolonged period after the peak of inflammation when EV quantity has already diminished. This underscores the potential of EV-lipid fingerprints as biomarkers of inflammation. Furthermore, the changes in the lipid fingerprint of EVs observed during inflammation might be indicative of their cells of origin. Among the cell types known to infiltrate the synovial cavity during inflammation, neutrophils are predominant, constituting up to 98% of cells during acute synovitis^[41]^. Based on the substantial increment in the number of neutrophils, the increase in HexCer in SF EVs might predominantly be attributed to neutrophil-derived EVs (nEVs).

## LIPID SIGNATURES OF EQUINE AND HUMAN NEUTROPHIL EVs

Previously, it has been shown that the release of EVs by neutrophils varies considerably depending on the activation status of these cells, independently of the secretion of primary or secondary granules, and that nEVs can act in a dualistic way, exhibiting pro- and anti-inflammatory properties^[122,123]^. Recently, we have demonstrated that the lipidome of EVs derived from unstimulated and fully activated equine neutrophils indeed showed a significant presence of HexCer, which was found to increase upon stimulation, accounting for nearly 50% of the total lipidome^[124]^. Additionally, human nEVs appeared to possess a significant proportion of glycosphingolipids, albeit Hex2Cer (dihexosylceramide), rather than HexCer, was identified in human nEVs as the predominant lipid^[124]^. This is in line with the observation made in the 1980s that a high proportion of Hex2Cer is present in human neutrophils, constituting approximately 70% of the total cellular glycosphingolipid content^[125,126]^. Unlike HexCer, Hex2Cer is composed of two hexose moieties, likely lactose, which consists of galactose and glucose subunits and is often referred to as lactosylceramide (LacCer)^[125,127]^. In human nEVs from unstimulated neutrophils, Hex2Cer is present, and its levels readily increase following stimulation, showing a stratified modification of the lipidome from nEVs derived from unstimulated neutrophils to single-stimulated and fully activated neutrophils^[124]^.

With respect to function, it has previously been suggested that Hex2Cer might play a central role in increasing the expression of CD11B/CD18, facilitating the generation of superoxide through its association with Src family kinase Lyn in lipid rafts, and triggering the activation of cytosolic phospholipase A2 (cPLA2), an enzyme that is vital for cleaving arachidonic acid-a precursor of prostaglandin-from phospholipids^[125,128,129]^. Furthermore, several pathogens have been found to bind to Hex2Cer, demonstrating its antibacterial properties^[130]^. HexCer is believed to be a by-product of the TNFα activation cascade, which can play a role in the immune response cascade, resulting in the activation of COX2^[131]^. Unfortunately, no other possible roles of HexCer have been studied extensively. Moreover, unlike human neutrophils, equine neutrophils require external arachidonic acid for the generation of leukotrienes^[132]^. This distinction might be attributed to the observed decreased activity of phospholipase A2 in equine neutrophils, which is less phosphorylated than in human neutrophils and equine eosinophils^[133]^. Considering the role of Hex2Cer in cPLA2 activation, it could be possible that the extra hexose distinguishing Hex2Cer from HexCer might be necessary to achieve the required structure for the production of leukotrienes without the external arachidonic acid, which equine neutrophils cannot produce autonomously. Importantly, despite many biological and molecular similarities among mammals^[134]^, subtle differences, such as the increased presence of HexCer or Hex2Cer in nEVs, could significantly impact translation from preclinical research to EV-based inflammatory biomarkers.

## NEUTROPHIL LIPID SIGNATURES IN HUMAN RHEUMATOID ARTHRITIS AND SPONDYLOARTHRITIS

Recently, we have demonstrated the presence of human nEV signatures in SF EVs of human patients with inflammatory arthropathies, i.e., rheumatoid arthritis (RA) and spondyloarthritis (SpA)^[124]^. Although these two conditions exhibit distinctive characteristics, both are universally recognized as autoimmune, systemic, and progressive diseases^[135,136]^. The discrimination between these diseases is often based on serological markers, the presence of rheumatoid factor (RF), and levels of antibodies against cyclic citrullinated peptides (anti-CCP), with the latter strongly supporting an RA diagnosis^[135]^. Based on the distinctions between RA and SpA, variations in the lipidome of the SF EVs might be anticipated. However, we did not observe significant differences in the lipidome of SF EVs derived from RA and SpA patients. In fact, there were many parallels^[124]^. This similarity can most likely be ascribed to the prominent influx of neutrophils into the SF, a feature both pathologies have in common^[32,137]^. Within the lipidome of SF EVs derived from RA and SpA patients, a substantial amount of Hex2Cer was detected, and the ratio of Hex2Cer to the entire lipidome resembled that of *in vitro* studies of human nEVs derived from partially stimulated neutrophils^[124]^. Remarkably, we recently showed that hyaluronic acid (HA) serves as a blocking agent of neutrophil activation, thereby implying a potential protective function of HA^[138]^. Based on these findings, we speculate that HA might partially block neutrophil activation *in vivo*, resulting in an nEV lipidome in SF that resembles the nEV lipidome of partially activated neutrophils *in vitro*^[124]^.

In SF EVs from SpA and RA patients, some HexCer species were also detected^[124]^. However, the relative abundance of HexCer did not come near to the lipid profile of SF EVs derived from the equine synovitis model^[121]^. A modest concentration of HexCer was also found in human neutrophil-derived EVs, but this relative abundance was too low to be considered the primary source for the levels observed in SF EVs from SpA and RA patients. Thus, the HexCer in SF EVs in these patients might originate from EVs derived from other cellular sources. Moreover, it was observed that also other lipid classes, including SM, PS, and PE, exhibited notably higher relative levels in SF from RA and SpA patients compared to the *in vitro* partially or fully stimulated nEVs from humans^[124]^. We propose that these differences might also be attributed to EVs derived from other cellular sources, potentially including T-cells and monocytes, among others.

Importantly, a more comprehensive understanding of the SF EV composition in relation to the cellular sources might serve as an EV-based diagnostic tool and might contribute to better endotype discovery in inflammatory joint diseases^[139]^.

## EVs AS COMPOSITE BIOMARKERS IN ARTHRITIS: AN EXAMPLE OF AN INTEGRATED OMICS APPROACH

In the clinical diagnosis of OA, emphasis is placed on symptoms such as pain, stiffness after inactivity, and functional limitations, and when necessary, supplementary diagnostic tools, such as radiographs, are employed^[140]^. However, patients with OA often display few symptoms in the early stages, leading to late diagnoses and making treatment increasingly challenging as the disease progresses. Furthermore, a better distinction in different molecular endotypes will be helpful for patient stratification and can avoid unnecessary treatments.

Recently, we conducted an exploratory study examining the proteome and lipidome composition of SF-derived EVs of horse patients with healthy joints as well as of those with naturally occurring mild and severe OA^[141]^. In contrast to the acute synovitis model in which a steep increase in the number of EVs was observed during acute inflammation, the impact of OA on the number of EVs in the SF was negligible, which has been corroborated by other studies^[53,54]^. Furthermore, the strong rise of HexCer in the EV-lipidome, as identified during acute synovitis, was not present in OA patient-derived SF EVs. Conversely, a relative increase in SM in mild OA was identified, with a more drastic increase in severe OA^[141]^. The proteomic analysis of EVs identified a functional enrichment of several pathways associated with disease phenotype, e.g., Rho family GTPases, including RAC family small GTPase1 and ezrin, were found to be activated in severe OA compared to healthy joints^[141]^. Hence, based on the analysis of the EV proteomes and lipidomes, a stratification pattern was observed, and, in line with the current view that all joint disorders, including OA, have an inflammatory component^[142]^, intensifying inflammation with disease progression was recognized^[141]^. Interestingly, the integration of phospholipidome and proteome data revealed potential composite biomarkers that exhibit a strong correlation, suggesting their potential utility for OA diagnosis and progression monitoring. Notable changes in downregulated proteins and phospholipids, including HSP90AA1 and CD163 with PC 34:4, PI 32:1, and PI 38:6, and upregulated proteins such as α-2 smooth muscle actin and ezrin, associated with SM 36:0;2, SM 41:3;2, and PC O-32:3, could serve as candidate composite SF-EV biomarkers for OA onset and progression^[141]^.

Since composite biomarkers better reflect the multifaceted nature of EVs than singular EV-biomarkers, multi-omics data integration of EV-associated molecules might be a novel approach for the identification of composite biomarkers in arthritis. Expanding the scope of biomarker discovery to encompass EVs at a higher and more comprehensive level by integrating lipid, protein, RNA, and metabolite analyses will strengthen the relevance of the findings. Such an approach could potentially sharpen diagnostic precision, allowing for subtle distinctions between indicators of a healthy joint versus those signaling the early onset of conditions such as arthritis and other diseases.

## CHALLENGES RELATED TO SF EV RESEARCH

In recent times, the field of EV research has witnessed significant advancements, with a flourishing community focusing on refining methodologies and steadily increasing the rigor of the science. The International Society for Extracellular Vesicles (ISEV) has been instrumental herein by disseminating numerous publications and taking initiatives aimed at enhancing knowledge about EVs^[143,144]^. Under the umbrella of ISEV, the Synovial Fluid Task Force was established. This Task Force aims to address the specific challenges in EV research concerning SF, including collection, handling, and storage of SF prior to EV isolation, as well as setting standards for experimentation and reporting, similar to what has been done with other ISEV task forces^[145-147]^. The ultimate goal is the standardization of procedures for SF-EV preparation to enhance consistency, reproducibility, and data comparability across research communities since SF, as a biofluid, presents a unique set of challenges, especially when sourced from inflamed joints. Our group and others have made efforts to find an efficient methodology for EV isolation from SF^[14,148]^. Nevertheless, obstacles remain, such as large aggregates that can be present in SF, especially from inflamed joints. Various SF pretreatments have been suggested, e.g., hyaluronidase, DNase, and anticoagulants such as sodium citrate, to avoid aggregation. At the moment, the SF Task Force is still in the process of defining guidelines for the improvement of EV preparation.

If unique SF EV composite markers can be defined, the next challenge would be to analyze whether these markers can also be detected in blood during active joint inflammatory diseases, which would make their potential use as biomarkers for disease prognosis and progression more feasible in a clinical setting. Furthermore, the profiling of other EV cargoes (e.g., proteins, RNA, metabolites) present in SF during inflammation in greater detail will fuel a more robust and comprehensive set of composite biomarkers for enhanced diagnostic procedures or for monitoring the effect of treatments.

## CONCLUSION

This review intends to highlight the knowledge gap regarding the lipidome of EVs, while also underscoring the potential of EV lipids as biomarkers and their role in enhancing our understanding of EV biogenesis and function. Lipidomics analysis of EVs has not only proven to be valuable but even necessary to better understand the molecular signatures of EVs in different pathological contexts, including arthritis. The distinct lipid signatures, e.g., the presence of HexCer in EVs during inflammation, can lead to the definition of novel biomarkers for diagnosing and monitoring inflammation related to arthritis. Additionally, comprehensive studies involving multi-omics integration of SF EVs have the potential to unravel composite biomarkers that can aid in better understanding disease heterogeneity and might be applied in clinical settings for diagnosis, monitoring disease progression, and tailoring treatments more precisely.

## DECLARATIONS

### Copyright

© The Author(s) 2024.
